# The Late Miocene Rifian corridor as a natural laboratory to explore a case of ichnofacies distribution in ancient gateways

**DOI:** 10.1038/s41598-021-83820-x

**Published:** 2021-02-18

**Authors:** Olmo Miguez-Salas, Francisco J. Rodríguez-Tovar, Wouter de Weger

**Affiliations:** 1 Departamento de Estratigrafía y Paleontología, Universidad de Granada, Avd. Fuentenueva s/n, 18002 Granada, Spain; 2 Department of Earth Sciences, Royal Holloway University of London, Egham, TW20 0EX Surrey UK

**Keywords:** Palaeoceanography, Palaeoclimate, Palaeoecology

## Abstract

Oceanic gateways have modulated ocean circulation and have influenced climatic variations throughout the Earth´s history. During the late Miocene (7.8–7.35 Ma), the Atlantic Ocean and the Mediterranean Sea were connected through the Rifian Corridor (Morocco). This gateway is one of the few examples of deep ancient seaways with a semi-continuous sedimentary record. Deposits comprise turbidites intercalated between deep-sea mudstone (i.e., hemipelagites and drift deposits), channelized sandstone contourite facies, and shallow marine sandstone. Herein an ichnological analysis was conducted in these upper Miocene sediments to improve characterisation of palaeoenvironmental conditions. In addition, ichnofacies were analysed to elucidate how bottom currents control ichnofacies distribution and can modify their attributes. Turbidite deposits are typified by vertical trace fossils (i.e., *Ophiomorpha*), conforming the *Ophiomorpha rudis* ichnosubfacies. Contouritic sandstone exhibits high density and low diversity trace-fossil assemblage, with predominant *Macaronichnus* and *Scolicia*, resembling a proximal expression of the *Cruziana* ichnofacies. Shallow marine environments are dominated by vertical trace fossils (e.g., *Conichnus*, *Ophiomorpha, Skolithos*), allowing an assignation to the *Skolithos* ichnofacies. This study reveals for the first time a variability in ichnofacies attributes and distribution at the Rifian Corridor, associated with turbidites, contourite and shallow marine sediments. Hydrodynamic energy reveals as the major factor controlling trace maker communities in the studied seaway. Highly energetic conditions typical of shallower settings are present in deeper-water environments (i.e., slope), contributing to ichnodiversity impoverishment in ichnofacies.

## Introduction

**Figure 1:**
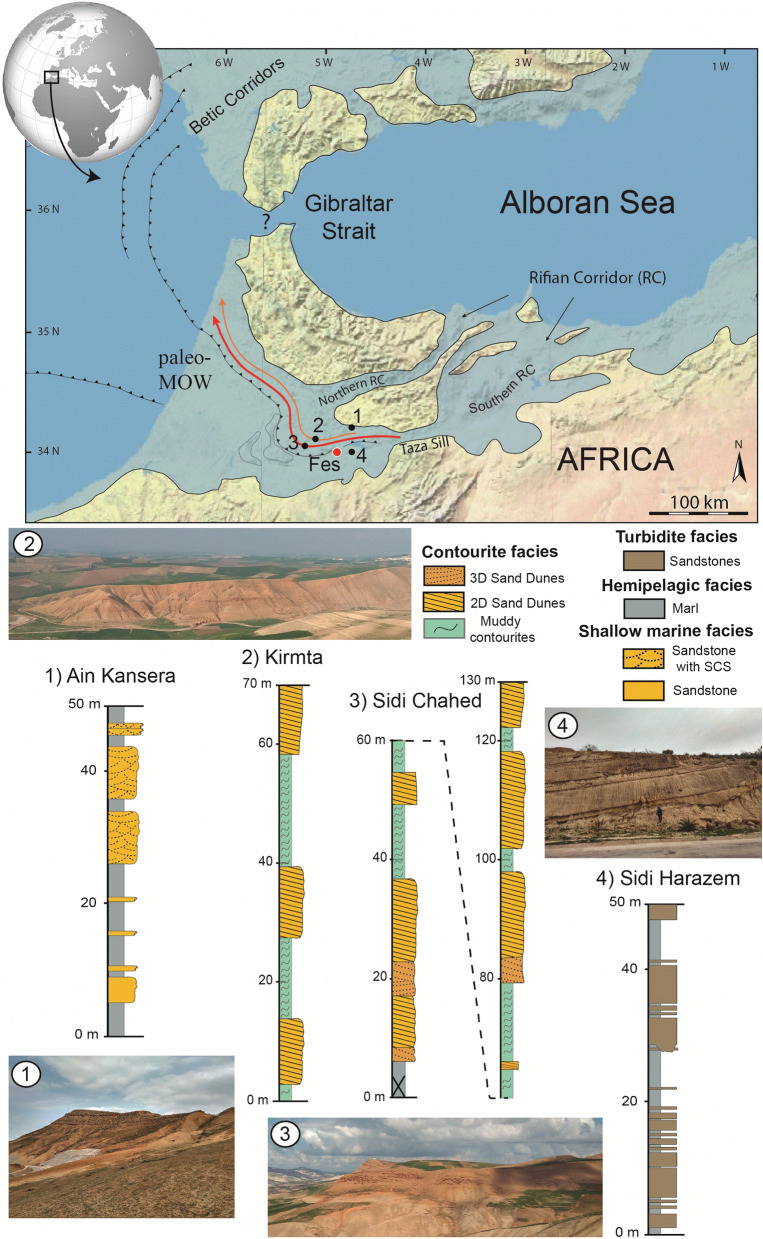
Palaeogeographic reconstruction of the late Miocene western Mediterranean with the location of the studied outcrops; red (lower) and orange (upper) arrows show palaeo-Mediterranean Outflow Water (palaeo-MOW) branches (modified from de Weger et al.^2^). Below, schematic sedimentary logs of the studied outcrops. Map created with Adobe Illustrator, version 22.1.0 (https://www.adobe.com/products/illustrator.html).

Oceanic gateways play a key role in controlling global ocean circulation and climate systems^1^. Ancient seaways are unique environments in which a complex interplay of processes may take place (i.e., oceanic-, tidal-, bottom-, turbiditic- and wind-currents)^2,3^. The constricted morphology of the seaway usually funnels and amplifies the currents that shape the seafloor (i.e., tidal currents)^4^. Previous sedimentological studies of ancient seaways have been largely focussed on shallow counterparts (generally between 100 and 150 m of water depth)^4–6^. Few published examples of deep ancient seaways (> 150 m) and associated deposits can be found. However, oceanographic studies have shown that deep seaways are different from shallow ones, with bottom-currents sometimes playing a dominant role^7–9^. The Rifian Corridor is one of those few examples (Fig. 1)^2,3,10,11^.

During the late Miocene, the Atlantic Ocean and the Mediterranean Sea were connected by two principal gateways, with a complex morphology, sills and channels through south Iberia and north Africa —the Betic and Rifian corridors, respectively^12,13^. The Rifian Corridor was a main deep seaway of this network (Fig. 1). This gateway progressively closed (7.1–6.9 Ma) due to tectonically induced uplift, leading to the onset of the Mediterranean Salinity Crisis in the late Miocene^13,14^. During the late Tortonian, the seaway evolved into a narrow, deep corridor hosting a complex interplay of processes^2,3^.

Ichnological analysis comprises a wide range of tools (e.g., ichnofabric approach, ichnofacies model) that prove very useful in sedimentary basin research^15^. The ichnofacies model is of special interest for detailed palaeoenvironmental reconstructions and for recognizing, distinguishing, and interpreting sedimentary environments^16–19^. Recent steps in ichnological research have established means of recognising and characterising contouritic processes, revealing the importance of ichnology as a proxy for discerning between contourites, turbidites, hemipelagites and pelagites^20–24^, but not without scepticism^25^. At any rate, the relationship between deep-sea settings and trace fossils is very complex, and depends highly upon the palaeonvironmental factors that affect trace makers^26^.

Trace-fossil research on seaway environments has been conducted mainly on shallow marine settings, including brackish-water ecosystems (i.e., estuarine complexes, resulting in the so-called “brackish-water model”^27,28^), beach–shoreface complexes with evidence of tidal processes^29,30^, and compound dune fields^31^. Still, detailed trace-fossil analysis and ichnofacies characterisation of ancient deep seaways has never been carried out. The aim of this research is to conduct a detailed ichnological analysis of selected outcrops of the Rifian Corridor (Ain Kansera, Sidi Chahed, Kirmta and Sidi Harazem), as a unique opportunity to assess trace-fossil variations to interpret an ancient deep-water seaway where shallow marine processes (i.e., tidal variations), pelagic/hemipelagic settling, turbiditic supplies and contouritic flows closely (less than 20 km) interact^2,3^. We evaluate the importance of palaeoenvironmental factors such as nutrients, oxygenation, and flow velocity in a setting dominated by bottom currents, and their incidence on the trace maker community. The utility of the ichnofacies approach is underlined within the framework of improving high-resolution palaeoenvironmental reconstructions in different depositional environments of ancient deep gateways.

### Trace-fossil assemblages at the Rifian Corridor

In both contouritic and turbiditic deposits, ichnodiversity is low (4 and 5 ichnogenera, respectively), whereas trace-fossil abundance is high in the former and moderate in the latter. Shallow marine deposits from the southern Rifian Corridor feature an abundant and moderately diverse trace-fossil assemblage (9 ichnogenera). Within the selected outcrops, the clear ichnological variability can be attributed to the different facies.

The Sidi Harazem turbiditic ichnoassemblage consists of 5 ichnogenera —*Ophiomorpha* (*O*. *rudis*), *Planolites*, *Spirophyton*, *Thalassinoides*, and *Zoophycos* (Fig. 3E–H)— and the thick sandstone beds are more bioturbated than the marly ones. *Ophiomorpha* is the most abundant ichnogenus, and appears in the thick turbiditic sandstone beds; *Thalassinoides* is common, *Planolites* rare, and *Zoophycos* and *Spirophyton* is occasionally found. The trace-fossil assemblage of marly pelagic and hemipelagic deposits from the Sidi Harazem consists of abundant undifferentiated structures and scarce *Planolites*-like and *Thalassinoides*-like trace fossils.

**Figure 2:**
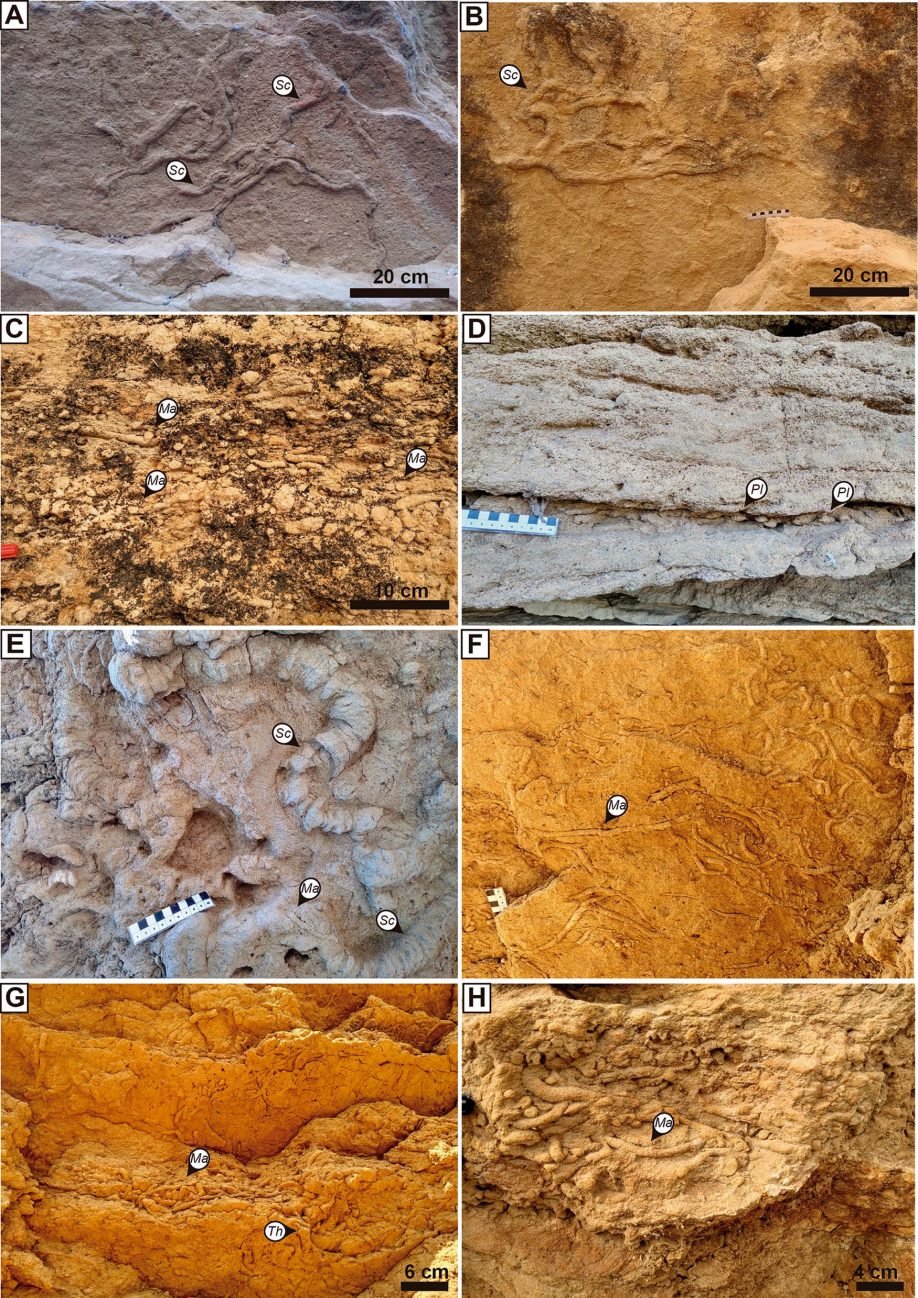
Trace-fossil specimens from the sandy contourite deposits at Sidi Chahed (**A**–**D**) and Kirmta (**E**–**H**) outcrops. (**A**, **B**) *Scolicia* in the sole of sandy clastic contouritic beds of Sidi Chahed; (**C**) Close-up view of *Macaronichnus* at Sidi Chahed; (**D**) *Planolites* within the interbedding of the foresets at Sidi Chahed. (**E**) *Scolicia* and some *Macaronichnus* at Kirmta; (**F**, **G**) *Macaronichnus* isp. and some *Thalassinoides* in the sole of sandy clastic contouritic beds at Kirmta; (**H**) Close-up view of *Macaronichnus* at Kirmta. *Macaronichnus* (*Ma*), *Planolites* (*Pl*), *Scolicia* (*Sc*), and *Thalassinoides* (*Th*).

The sandy contourites in Kirmta and Sidi Chahed comprise a highly abundant and scarcely diverse trace-fossil assemblage (4 ichnogenera), dominated by *Macaronichnus* and *Scolicia*, and common *Planolites* and *Thalassinoides* (Fig. 2). Trace fossils were predominantly found in the planar-stratified and cross-bedded sandstone. Turbidites show an absence of discrete trace fossils. The trace = fossil assemblage of muddy contourite deposits from both outcrops consist of regular undifferentiated biogenic structures and scarce *Planolites*-like and *Thalassinoides*-like trace fossils.

**Figure 3:**
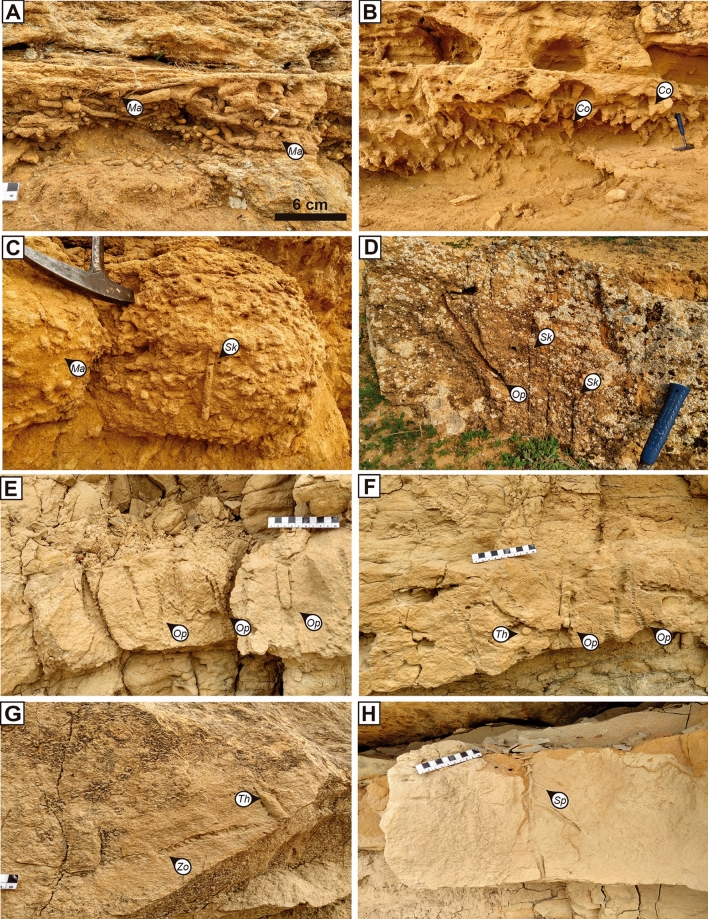
Trace-fossil specimens from shallow marine deposits at Ain Kansera (**A**–**D**) and turbiditic deposits at Sidi Harazem (**E**–**H**). (**A**) Close-up view of *Macaronichnus* at Ain Kansera; (**B**) Densely *Conichnus* assemblage at Ain Kansera; (**C**) *Macaronichnus* cross-cut by a *Skolithos* at Ain Kansera; (**D**) *Skolithos* and *Ophiomorpha* at Ain Kansera; (**E**, **F**) *Ophiomorpha* (*O. rudis*) at Sidi Harazem; (**G**) *Zoophycos* cross-cut by a *Thalassinoides* at Sidi Harazem; (**H**) Close-up view of *Spyrophyton* at Sidi Harazem. *Conichnus* (*Co*), *Macaronichnus* (*Ma*), *Ophiomorpha* (*Op*), *Skolithos* (*Sk*), *Spyrophyton* (*Sp*), *Thalassinoides* (*Th*), and *Zoophycos* (*Zo*).

The Ain Kansera section is characterised by a shallow marine ichnoassemblage with high ichnodiversity and an abundance of vertical structures, including 9 ichnogenera in the sandstone beds: *Conichnus*, *Diplocraterion*, *Macaronichnus*, *Ophiomorpha*, *Parahaentzschelinia*, *Planolites*, *Scolicia*, *Skolithos*, and *Thalassinoides* (Fig. 3A–D). The sandstone beds with swaley cross-stratification show a change in the trace-fossil assemblage towards the top of the outcrop. The lower sandstone beds present dominant *Conichnus* and *Macaronichnus*, common *Parahaentzschelinia* and *Thalassinoides*, and rare *Diplocraterion*, *Planolites*, and *Scolicia*. The upper sandstone beds record the disappearance of *Conichnus* and *Parahaentzschelinia*, while *Ophiomorpha* and *Skolithos* become dominant.

### Ichnofacies characterisation

The trace-fossil assemblage of Sidi Harazem is typified by vertical burrows of *Ophiomorpha rudis* and some *Thalassinoides*. *Ophiomorpha* is generally but not exclusively characteristic of high-energy environments (i.e., shoreface) in well-sorted, shifting sandy substrates, constituting a common element of the *Skolithos* and *Cruziana* ichnofacies^17,18^. However, the appearance of *Ophiomorpha* in deep-sea environments is also recorded, and usually explained as an effect of transport of the trace makers by currents from shallow marine environments into the deep-sea^33,34^. Uchman^35^ proposed the *Ophiomorpha rudis* ichnosubfacies within the *Nereites* ichnofacies for the record of ichnoassemblages dominated by *Ophiomorpha rudis* in thick sandstone beds related with channels and proximal lobes in turbiditic systems^36^. Accordingly, the Sidi Harazem trace-fossil assemblage could be associated with the *Ophiomorpha rudis* ichnosubfacies. Ichnosubfacies/ichnofacies assignation is tentative due to the absence of other components of this ichnosubfacies (e.g., *Scolicia*, *Nereites*, graphoglyptids); this uncertainty is tied to outcrop limitations, e.g. the low exposure of turbiditic soles and difficulties in observing discrete trace fossils in the non-compact hemipelagic and pelagic deposits.

The trace-fossil assemblages of Kirmta and Sidi Chahed feature high abundance and low ichnodiversity, being dominated by horizontal trace fossils, such as *Macaronichnus* and *Scolicia*. *Macaronichnus* is usually interpreted as a shallow marine (up to foreshore) trace fossil^37^ that occasionally appears in deeper water environments^38,39^ and is commonly associated with the *Skolithos* ichnofacies^17–19,40^. *Scolicia* presents a wide environmental range, but is a typical element of the deep-marine *Nereites* and the shelfal *Cruziana* ichnofacies^40^. The proximal expression of the *Cruziana* ichnofacies is dominated by deposit-feeding burrows, but also includes structures of passive carnivores, omnivores, suspension feeders, as well as grazing forms^41^. This ichnofacies is defined as a transition between the distal expression of the *Skolithos* ichnofacies and the archetypal *Cruziana* ichnofacies^41^. The low ichnodiversity observed within the contourite facies from Kirmta and Sidi Chahed outcrops, together with the ubiquity of the dominant trace fossils, hamper a conclusive ichnofacies assignation. Still, though *Macaronichnus* is typical from high energy shallow marine environments, it may locally appear in the proximal *Cruziana* ichnofacies^41^. Considering the dominance of horizontal feeding trace fossils produced by deposit and detritus feeders over dwelling structures of suspension feeding structures, contourite ichnoassemblages at the Rifian Corridor, registered at Kirmta and Sidi Chahed outcrops, can therefore be tentatively assigned to an impoverished proximal *Cruziana* ichnofacies^18^.

The trace-fossil assemblage of Ain Kansera is characterised by moderate ichnodiversity with a dominance of vertical (*Skolithos* and *Ophiomorpha*), cylindrical or conic-shaped (*Conichnus*) dwelling burrows of suspension feeders and passive predators. Horizontal trace fossils produced by a mobile fauna are scarce, mainly associated with *Macaronichnus* trace makers. According to these ichnological features, shallow marine facies at the Rifian Corridor —represented by Ain Kansera sediments— can be clearly assigned to the *Skolithos* ichnofacies, with predominant burrow systems having vertical, cylindrical, or U-shaped components of suspension feeders and passive predators, and a scarcity of horizontal trace fossils^17–19,40,42^.

### Ichnofacies in the Rifian Corridor seaways: hydrodynamic energy and the incidence of bottom currents

Over the past years, detailed ichnological research has revealed the major incidence of particular environmental factors (e.g., organic-matter content, oxygenation, sedimentation rate) on ichnological attributes from deep-sea environments, including ichnofacies characterisation and distribution^26^. The deep sea is a complex environment where several depositional processes co-exist, including pelagic/hemipelagic settling, bottom currents and gravity flows^9^. Trace-fossil analysis has proven useful for discerning and characterising such sedimentary environments and associated deposits^21^. Hydrodynamic conditions are a very significant limiting factor for trace makers, inducing variations in distribution and behaviour, hence in the preservation of trace fossils^19,29,43,44^. Typically, ichnoassemblages related to high energy conditions are characterised by vertical dwelling structures of infaunal suspension feeders and/or passive predators, forming low-diversity suites; ichnoassemblages related to low energy conditions are dominated by horizontal feeding trace fossils of deposit and detritus feeders, as well as higher diversity^19^. Ichnofacies identification is mainly based on the recognition of key features that connect biological structures with physical parameters (i.e., environmental conditions)^17–19^. Accordingly, ichnofacies reflect specific combinations of organisms´ responses to a wide range of environmental conditions.

**Figure 4:**
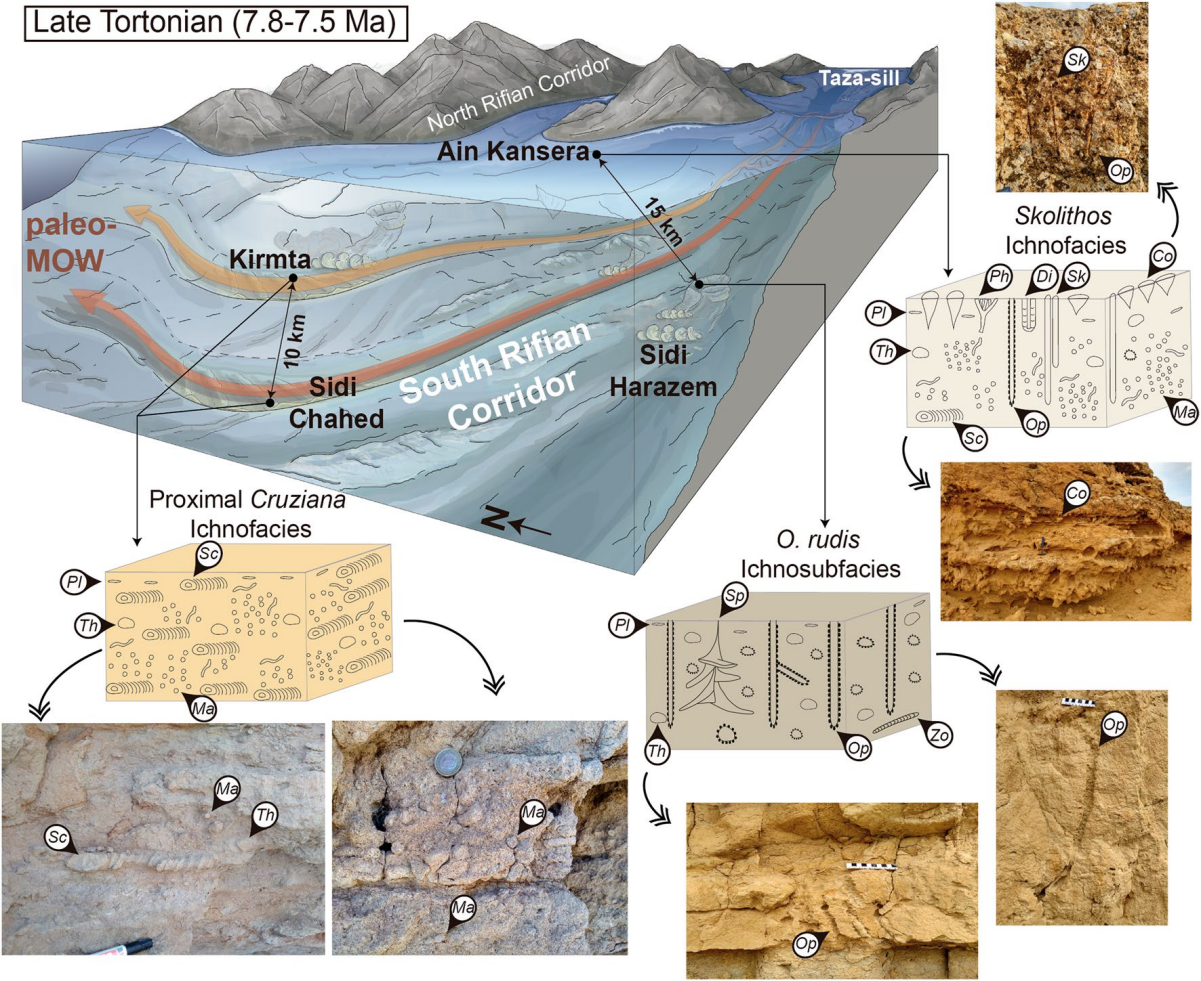
Palaeogeographic model of the late Miocene Rifian Corridor (Morocco) with ichnofacies distribution (lower red and upper orange branches indicate palaeo-MOW location; modified from de Weger et al.^2^). * Conichnus* (*Co*), *Diplocraterion* (*Di*), *Macaronichnus* (*Ma*), *Ophiomorpha* (*Op*), *Parahaentzschelinia* (*Ph*), *Planolites* (*Pl*), *Scolicia* (*Sc*), *Skolithos* (*Sk*), *Spyrophyton* (*Sp*), *Thalassinoides* (*Th*), and *Zoophycos* (*Zo*).

In the case of seaways, prevailing hydrodynamic conditions are a main environmental factor, along with controlling depositional processes and sedimentation regimes^6,30^. Even though the number of trace-fossil studies is considerably lower than in other clastic shallow or deep marine environments, ichnological analysis has proven to be useful to characterise waves, tides or storms in shallow seaways^29,30^, overlooking deep seaways and their implications. Deep seaways with narrow palaeogeographical configuration, as is the case of the Rifian Corridor^10^, would promote higher energetic conditions than those typical of deep-sea environments. In the study area, clearly distinct sedimentary environments —in terms of hydrodynamic conditions, bathymetry, rate of sedimentation, etc.— are closely spaced^2^, passing from shallow marine to turbiditic slope systems in less than 20 km (Fig. 4). Such variations in palaeoenvironmental conditions are supported by ichnofacies characterisation and distribution.

Turbidite deposits from Sidi Harazem, emplaced on the slope of the Rifian Corridor, are typified by vertical trace fossils, mainly by the record of *Ophiomorpha rudis*. These ichnological attributes are similar to those associated with particular sub-environments (e.g., channels and proximal trubiditic lobes) of the turbiditic systems, conforming the *Ophiomorpha rudis* ichnosubfacies inside the *Nereites* ichnofacies^36^.

Sandy contourite 2D- and 3D-dune facies (upper slope environment) (Fig. 4) from Sidi Chahed and Kirmta are related to high-energy deep-water environments. However, they are dominated by horizontal trace fossils (*Macaronichnus* and *Scolicia*) produced by mobile deposit- and detritus-feeders, discarding a direct assignation to the *Skolithos* ichnofacies. In this case, palaeoenvironmental conditions other than hydrodynamic energy must be considered to explain the dominance of horizontal forms and the absence of vertical biogenic structures. The record of densely *Macaronichnus* ichnoasemblages in these contourite sediments was recently linked to high nutrient supply provided by ancient bottom currents^39,45^. This agrees with the record of *Scolicia*: its abundance and size usually increase in conjunction with greater amounts and nutritious values of benthic food^20,46^. Thus, the strong palaeo-MOW bottom currents that dominated the slope may have created well-oxygenated and nutrient-rich benthic environments, favouring colonisation by trace makers that could exploit such accumulations of organic matter inside the sediment. *Macaronichnus* and *Scolicia* producers could develop an opportunistic behaviour, determining rapid and complete bioturbation, avoiding colonisation by other trace makers —including suspension feeders—these ichnological features resemble the *Cruziana* ichnofacies attributes. Notwithstanding, the high ichnodiversity that is characteristic of the *Cruziana* ichnofacies is absent here. The great abundance and low ichnodiversity observed for the contourite facies appear to indicate the absence of an archetypal *Cruziana* ichnofacies, but the development of the proximal *Cruziana* ichnofacies. Bottom currents and their associated deposits (i.e., contourites) have been previously linked to both the *Cruziana* and *Zoophycos* ichnofacies in Cyprus Miocene carbonate contourite deposits^22,23^, meaning that contourite deposits are not exclusively related to a single ichnofacies. The replacement from the *Zoophycos* to *Cruziana* ichnofacies was interpreted to be mainly controlled by sea level dynamics^23^.

The shallow marine facies from Ain Kansera (shoreface environment) are dominated by vertical, cylindrical, or U-shaped dwelling burrows (*Conichnus*, *Ophiomorpha* and *Skolithos*) of suspension feeders (Fig. 4). These attributes are usually related to high energetic conditions developed in shallow marine environments conforming the *Skolithos* ichnofacies^18^.

In short, at the Rifian Corridor, ichnofacies distributions from proximal to distal settings are controlled by bottom currents (palaeo-MOW), with hydrodynamic conditions being the major palaeonvironmental limiting factor. Particularly noteworthy is the development of the proximal *Cruziana* ichnofacies in deeper settings from the slope environments; bottom currents generated high energetic conditions similar to those of shallow/proximal areas.

## Conclusions

During the late Miocene, the Rifian Corridor (Morocco) connected the Atlantic Ocean and the Mediterranean Sea. The particular palaeogeographical configuration led this ancient deep seaway to be affected by variable palaeoceanographic processes and associated deposits (e.g., shallow marine sandstones, channelized sandstone contourite facies, and turbidites intercalated between deep-sea hemipelagites), inducing different ichnological features in terms of ichnofacies composition and distribution. Turbidite deposits are typified by vertical trace fossils (i.e., *Ophiomorpha*), with assignation to the *Ophiomorpha rudis* ichnosubfacies. Contourite deposits record ichnological assemblages dominated by *Scolicia* and *Macaronichnus*, with a plausible assignation to the impoverished proximal *Cruziana* ichnofacies. Shallow marine environments are dominated by vertical trace fossils (e.g., *Conichnus*, *Ophiomorpha, Skolithos*) conforming the *Skolithos* ichnofacies. This research evidenced that ichnofacies distribution in the studied ancient seaway (Rifian Corridor) is mainly controlled by the prevailing hydrodynamic regimes.

## Methods

### Geological setting

The Rifian Corridor (Morocco) connected the Atlantic Ocean and the Mediterranean Sea during the late Miocene (Fig. 1). The outcrops studied herein are located on the northern flank of the Saiss Basin in the South Rifian Corridor, west of Taza-sill^10^ (Fig. 1). This basin comprises middle to upper Miocene foreland deposits^32^ and records a unique contourite channel system related to the palaeo-Mediterranean Outflow Water (Palaeo-MOW^2^), which resulted from net evaporation in the Mediterranean leading to dense water formation. Regional tectonic activity favoured the development of turbiditic and gravity flows, inducing a complex deep system in which variable sedimentary processes interacted^2^.

Four outcrops (Fig. 1) from the Saiss Basin were selected for study: (1) Sidi Harazem (34°01′52.67″N, 4°52′47.69″W), (2) Kirmta (34°10′15.07″N, 5°14′21.43″W) (3) Sidi Chahed (34°05′58.07″N, 5°18′15.12″W), and (4) Ain Kansera (34°07′34.06″N, 4°51′20.04″W). A brief facies description is included below, for more detailed information see Capella et al.^10^ and de Weger et al.^2^.

The Sidi Harazem outcrop consists mainly of upper Tortonian (between 7.80 and 7.51 Ma) sandstone and marlstone intercalations^10^. The sandstone beds, up to 4 m thick, are commonly structureless, normally graded, and composed of poorly to moderately sorted, medium- to coarse-grained sand. Locally, channel-like features are observed. The deposits have been associated to a basinal turbidite system with benthic foraminiferal assemblages indicating water depths of 250–400 m^10^.

The Sidi Chahed sections, previously studied by Capella et al.^10^ and de Weger et al.^2^, and the Kirmta outcrop, previously studied by de Weger et al.^2^, consist of three main channelized upper Tortonian (7.8–7. 51 Ma) sandstone units encased in blue marls. Both outcrops contain different orders of unidirectional traction structures, ranging from ripples to m-scale 3D-dunes. Interbedded slump deposits have been identified within the main sandstone bodies, while turbidites have been recognized between the main sandstone bodies that are encased by marlstones^2^. In both outcrops, the blue marls have been most likely interpreted as muddy contourites (see Capella et al.^10^ and de Weger et al.^2^). Both outcrops were deposited within a palaeodepth range from the upper slope to the outer shelf (150–400 m water depth)^2,10^.

The Ain Kansera outcrop consists of upper Tortonian (between 7.51 and 7.31 Ma) sandstone and marlstone intercalations^10^. Sandstone bed thicknesses range from 1 to 10 m, the beds containing medium- to coarse- and very coarse-grained sand. They consist of a compositional mix of siliciclastic and bioclastic sand, and regularly contain hummocky and swaley cross-stratification. Benthic foraminiferal assemblages and the presence of hummocky cross-stratification indicate an inner shelf environment (water depths of 50–100 m)^10^. Toward the top, the thick sandstone intervals probably represent a shallower (15–50 m water depth) wave-dominated infralittoral setting.

### Methodology

Ichnological analysis from the selected sections at Sidi Harazem (50 m), Sidi Chahed (130 m), Kirmta (70 m) and Ain Kansera (50 m) was conducted first at the outcrop and then in laboratory. Special attention was pay to the correlation between ichnological attributes, facies and bed features (preservation within sedimentary beds and relationship with bottom and top surfaces). Outcrop analysis was performed bed-by-bed focusing on abundance, diversity and macroscopic morphological burrow features (e.g., orientation, shape, length/diameter, cross-cutting relationships, and taphonomy). Collected specimens were examined in the laboratory under microscope, with special attention to ichnotaxonomical features (e.g., infilling material, burrow wall). The images of some photographed specimens (outcrop and laboratory) were treated with image software to improve visibility of ichnological features^47^. Collected samples are housed in the Department of Stratigraphy and Palaeontology, University of Granada.

## Data Availability

All data analysed in this study are summarised in this published article. The original datasets are not publicly available due to size restrictions, but are available from the corresponding author upon request.

## References

[1] Knutz PC, Rebesco M, Camerlenghi A (2008). Paleoceanographic significance of contourite drifts. Contourites.

[2] de Weger W, Hernández-Molina FJ, Flecker R, Sierro FJ, Chiarella D, Krijgsman W, Amine Manar M (2020). Late Miocene contourite channel system reveals intermittent overflow behavior. Geology.

[3] de Weger, W., Hernández-Molina, F. J., Miguez-Salas, O., de Castro, S., Bruno, M., Chiarella, D., Sierro, F. J., Blackbourn, G. & Manar, M. A. Sedimentary evolution of a laterally migrating contourite depositional system—a case study from the late Miocene Rifian Corridor, Morocco. *Sedimentology*. (submitted).

[4] Longhitano SG (2013). A facies-based depositional model for ancient and modern, tectonically-confined tidal straits. Terra Nova.

[5] Anastas AS, Dalrymple RW, James NP, Nelson CS (2006). Lithofacies and dynamics of a cool-water carbonate seaway: mid-Tertiary, Te Kuiti Group, New Zealand. Geol. Soc. Spec. Publ..

[6] Olariu C, Steel RJ, Dalrymple RW, Gingras MK (2012). Tidal dunes versus tidal bars: the sedimentological and architectural characteristics of compound dunes in a tidal seaway, the lower Baronia Sandstone (Lower Eocene), Ager Basin, Spain. Sed. Geol..

[7] Legg S, Chang Y, Chassignet E, Danabasoglu G, Ezer T, Gordon A, Griffes S, Hallberg R, Jackson L, Large W, Ozgokmen T, Peters H, Price J, Riemenschneider U, Wu W, Xu X, Yang J (2009). Improving oceanic overflow representation in climate models: the Gravity Current Entrainment Climate Process Team. Bull. Am. Meteorol. Soc..

[8] Hernández-Molina FJ, Llave E, Preu B, Ercilla G, Fontan A, Bruno M, Serra N, Gomiz JJ, Brackenridge RE, Sierro FJ, Stow DAV, García M, Juan C, Sandoval N, Arnaiz A (2014). Contourite processes associated with the Mediterranean Outflow Water after its exit from the Strait of Gibraltar: Global and conceptual implications. Geology.

[9] Rebesco M, Hernández-Molina FJ, Van Rooij D, Wåhlin A (2014). Contourites and associated sediments controlled by deep-water circulation processes: state of the art and future considerations. Mar. Geol..

[10] Capella W, Hernández-Molina FJ, Flecker R, Hilgen FJ, Hssain M, Kouwenhoven TJ, van Oorschot M, Sierro FJ, Stow DAV, Trabucho-Alexandre J, Tulbure MA (2017). Sandy contourite drift in the late Miocene Rifian Corridor (Morocco): reconstruction of depositional environments in a foreland-basin seaway. Sed. Geol..

[11] Capella W, Barhoun N, Flecker R, Hilgen FJ, Kouwenhoven T, Matenco LC, Sierro FJ, Tulbure MA, Yousfi MZ, Krijgsman W (2018). Palaeogeographic evolution of the late Miocene Rifian Corridor (Morocco): reconstructions from surface and subsurface data. Earth Sci. Rev..

[12] Flecker R, Krijgsman W, Capella W, de Castro Martíns C, Dmitrieva E, Mayser JP, Marzocchi A, Modestou S, Ochoa D, Simon D, Tulbure M (2015). Evolution of the Late Miocene Mediterranean–Atlantic gateways and their impact on regional and global environmental change. Earth Sci. Rev..

[13] Krijgsman W, Capella W, Simon D, Hilgen FJ, Kouwenhoven TJ, Meijer PT, Sierro FJ, Tulbure MA, van den Berg BC, van der Schee M, Flecker R (2018). The Gibraltar corridor: watergate of the Messinian salinity crisis. Mar. Geol..

[14] Capella W, Matenco L, Dmitrieva E, Roest WM, Hessels S, Hssain M, Chakor-Alamic A, Sierro FJ, Krijgsman W (2017). Thick-skinned tectonics closing the Rifian Corridor. Tectonophysics.

[15] Knaust D, Bromley RG (2012). Trace Fossils as Indicators of Sedimentary Environments.

[16] Frey RW, Pemberton SG, Saunders TD (1990). Ichnofacies and bathymetry: a passive relationship. J. Pal..

[17] MacEachern, J. A., Pemberton, S. G., Gingras, M. K. & Bann, K. L. The ichnofacies paradigm: a fifty-year retrospective. in *Trace fossils* (ed. Miller III, W.), 52–77. (Elsevier, 2007).

[18] MacEachern JA, Bann KL, Gingras MK, Zonneveld JP, Dashtgard SE, Pemberton SG, Knaust D, Bromley RG (2012). The ichnofacies paradigm. Trace Fossils as Indicators of Sedimentary Environments.

[19] Buatois LA, Mángano MG (2011). Ichnology: Organism–Substrate Interactions in Space and Time.

[20] Wetzel A, Werner F, Stow DAV, Rebesco M, Camerlenghi A (2008). Bioturbation and biogenic sedimentary structures in contourites. contourites.

[21] Rodríguez-Tovar FJ, Hernández-Molina FJ (2018). Ichnological analysis of contourites: past, present and future. Earth Sci. Rev..

[22] Miguez-Salas O, Rodríguez-Tovar FJ (2019). Stable deep-sea macrobenthic trace maker associations in disturbed environments from the Eocene Lefkara Formation, Cyprus. Geobios.

[23] Miguez-Salas O, Rodríguez-Tovar FJ (2019). Ichnofacies distribution in the Eocene–early Miocene Petra Tou Romiou outcrop, Cyprus: sea level dynamics and palaeoenvironmental implications in a contourite environment. Int. J. Earth Sci..

[24] Rodríguez-Tovar FJ, Hernández-Molina FJ, Hüneke H, Chiarella D, Llave E, Mena A, Miguez-Salas O, Dorador J, De Castro S, Stow DAV (2019). Key evidence for distal turbiditic-and bottom-current interactions from tubular turbidite infills. Palaeogeogr. Palaeoclimatol. Palaeoecol..

[25] Shanmugam G, Mazumder R (2017). The contourite problem. Sediment Provenance.

[26] Wetzel A, Uchman A, Knaust D, Bromley RG (2012). Hemipelagic and pelagic basin plains. Trace Fossils as Indicators of Sedimentary Environments.

[27] MacEachern, J. A. & Gingras, M. K. Recognition of brackish-water trace fossil suites in the Cretaceous Western Interior Seaway of Alberta, Canada. In *Sediment-Organism Interactions: A Multifaceted Ichnology* (eds. Bromley, R. G., Buatois, L. A., Mángano, G., Genise, J .F. & Melchor, R. N.), 50–59. (SEPM Spec. Publ. 2007).

[28] Angulo S, Buatois LA (2012). Ichnology of a Late Devonian-Early Carboniferous low-energy seaway: the Bakken Formation of subsurface Saskatchewan, Canada: Assessing palaeoenvironmental controls and biotic responses. Palaeogeogr. Palaeoclimatol. Palaeoecol..

[29] Frey SE, Dashtgard SE (2011). Sedimentology, ichnology and hydrodynamics of strait-margin, sand and gravel beaches and shorefaces: Juan de Fuca Strait, British Columbia, Canada. Sedimentology.

[30] Colella A, d'Alessandro A (1988). Sand waves, Echinocardium traces and their bathyal depositional setting (Monte Torre Palaeostrait, Plio-Pleistocene, southern Italy). Sedimentology.

[31] Desjardins PR, Buatois LA, Pratt BR, Mangano MG (2012). Sedimentological–ichnological model for tide-dominated shelf sandbodies: Lower Cambrian Gog Group of western Canada. Sedimentology.

[32] Sani F, Del Ventisette C, Montanari D, Bendkik A, Chenakeb M (2007). Structural evolution of the Rides Prerifaines (Morocco): structural and seismic interpretation and analogue modelling experiments. Int. J. Earth Sci..

[33] Wetzel, A. Bioturbation in deep-sea fine-grained sediments: influence of sediment texture, turbidite frequency and rates of environmental changes. in *Fine Grained Sediments: Deep-Water Processes and Facies*. (eds. Stow, D. A. V. & Piper, D. J. W.), 597–608. (Geological Society of London, Special Publication, 1984).

[34] Föllmi KB, Grimm KA (1990). Doomed pioneers: gravity-flow deposition and bioturbation in marine oxygen-deficient environments. Geology.

[35] Uchman A (2001). Eocene flysch trace fossils from the Hecho Group of the Pyrenees, northern Spain. Beringeria.

[36] Uchman A (2009). The *Ophiomorpha rudis* ichnosubfacies of the *Nereites* ichnofacies: characteristics and constraints. Palaeogeogr. Palaeoclimatol. Palaeoecol..

[37] Seike K (2007). Palaeoenvironmental and palaeogeographical implications of modern *Macaronichnus segregatis*-like traces in foreshore sediments on the Pacific coast of Central Japan. Palaeogeogr. Palaeoclimatol. Palaeoecol..

[38] Rodríguez-Tovar FJ, Aguirre J (2014). Is Macaronichnus an exclusively small, horizontal and unbranched structure? Macaronichnus segregatis degiberti isubsp. nov. Span. J. Paleontol..

[39] Miguez-Salas O, Rodríguez-Tovar FJ, De Weger W (2020). *Macaronichnus* and contourite depositional settings: bottom currents and nutrients as coupling factors. Palaeogeog. Palaeocl. Palaeoecol..

[40] Knaust D (2017). Atlas of Trace Fossils in Well Core.

[41] MacEachern, J. A. & Bann, K. L. The role of ichnology in refining shallow marine facies models. in *Recent Advances in Models of Siliciclastic Shallow-Marine Stratigraphy* (eds. Hampson, G. J., Steel, R. J., Burgess, P. B. & Dalrymple, R. W.), 73–116 (SEPM Spec. Publ. 2008)

[42] Pemberton, S. G., van Wagoner, J. G. & Wach, G. D., Ichnofacies of a wave dominated shoreline. in *Application of Ichnology to Petroleum Exploration* ( ed. Pemberton, S. G.), 339–382. (Society of Economic Paleontologists and Mineralogists, Core Workshop Notes, 1992).

[43] Anderson BG, Droser ML (1998). Ichnofabrics and geometric configurations of *Ophiomorpha* within a sequence stratigraphic framework: an example from the Upper Cretaceous US western interior. Sedimentology.

[44] Dorador J, Rodríguez-Tovar FJ, Mena A, Francés G (2019). Lateral variability of ichnological content in muddy contourites: weak bottom currents affecting organisms’ behavior. Sci. Rep..

[45] Miguez-Salas O, Rodríguez-Tovar FJ (2020). Trace fossil analysis of sandy clastic contouritic deposits in the late Miocene Rifian Corridor (Morocco): Ichnotaxonomical and palaeoenvironmental insights. J. Afr. Earth Sci..

[46] Wetzel A (2008). Recent bioturbation in the deep South China Sea: a uniformitarian ichnologic approach. Palaios.

[47] Miguez-Salas O, Dorador J, Rodríguez-Tovar FJ (2019). Introducing Fiji and ICY image processing techniques in ichnological research as a tool for sedimentary basin analysis. Mar. Geol..

